# Genomic Instability and *TP53* Genomic Alterations Associate With Poor Antiproliferative Response and Intrinsic Resistance to Aromatase Inhibitor Treatment

**DOI:** 10.1200/PO.18.00286

**Published:** 2019-06-12

**Authors:** Eugene F. Schuster, Pascal Gellert, Corrinne V. Segal, Elena López-Knowles, Richard Buus, Maggie Chon U. Cheang, James Morden, John Robertson, Judith M. Bliss, Ian Smith, Mitch Dowsett, on behalf of the POETIC Trial Management Group and Trialists

**Affiliations:** ^1^ The Institute of Cancer Research, London, United Kingdom; ^2^ Royal Marsden Hospital, London, United Kingdom; ^3^ University of Nottingham, Nottingham, United Kingdom

## Abstract

**PURPOSE:**

Although aromatase inhibitor (AI) treatment is effective in estrogen receptor–positive postmenopausal breast cancer, resistance is common and incompletely explained. Genomic instability, as measured by somatic copy number alterations (SCNAs), is important in breast cancer development and prognosis. SCNAs to specific genes may drive intrinsic resistance, or high genomic instability may drive tumor heterogeneity, which allows differential response across tumors and surviving cells to evolve resistance to treatment rapidly. We therefore evaluated the relationship between SCNAs and intrinsic resistance to treatment as measured by a poor antiproliferative response.

**PATIENTS AND METHODS:**

SCNAs were determined by single nucleotide polymorphism array in baseline and surgery core-cuts from 73 postmenopausal patients randomly assigned to receive 2 weeks of preoperative AI or no AI in the Perioperative Endocrine Therapy—Individualizing Care (POETIC) trial. Fifty-six samples from the AI group included 28 poor responders (PrRs, less than 60% reduction in protein encoded by the *MKI67* gene [Ki-67]) and 28 good responders (GdRs, greater than 75% reduction in Ki-67). Exome sequencing was available for 72 pairs of samples.

**RESULTS:**

Genomic instability correlated with Ki-67 expression at both baseline (*P* < .001) and surgery (*P* < .001) and was higher in PrRs (*P* = .048). The SCNA with the largest difference between GdRs and PrRs was loss of heterozygosity observed at 17p (false discovery rate, 0.08), which includes *TP53.* Nine of 28 PrRs had loss of wild-type *TP53* as a result of mutations and loss of heterozygosity compared with three of 28 GdRs. In PrRs, somatic alterations of *TP53* were associated with higher genomic instability, higher baseline Ki-67, and greater resistance to AI treatment compared with wild-type *TP53*.

**CONCLUSION:**

We observed that primary tumors with high genomic instability have an intrinsic resistance to AI treatment and do not require additional evolution to develop resistance to estrogen deprivation therapy.

## INTRODUCTION

Estrogen deprivation is the major treatment strategy for hormone-dependent breast cancer (BC) and typically involves agents that inhibit aromatase, the enzyme that catalyzes the conversion of androgens to estrogens. Despite near-complete suppression of circulating estrogen levels by aromatase inhibitor (AI) treatment, acquired and de novo resistance to AI is common.^1^ Few pretreatment biomarkers exist for AI resistance, and mechanisms of resistance are incompletely understood.^2^

Mutations and somatic copy number alterations (SCNAs) can play important roles in activating oncogenes or inactivating tumor suppressors, and BC is characterized by multiple recurrent SCNAs and few recurrent mutations.^3^ We have previously shown that *TP53* mutations (*TP53*^MUT^) occur at a higher rate in tumors with poor response to AI treatment, which suggests that these patients received less benefit from AI^4^ but that SCNAs to specific genes also may play an important role in AI resistance.^5^ Nonspecific genomic alterations, like high genomic instability, are known to be associated with poor prognosis and probably at least partly the result of tumor heterogeneity, which allows some cells to survive and evolve resistance to treatment.^6^ There is evidence in other solid tumors of an association between high genomic instability and intrinsic resistance to chemotherapy.^7^ However, few studies of genomic instability and response to endocrine treatment exist. The aim of the current work, therefore, was to determine whether genome-wide measures of SCNAs (ie, genomic instability) and/or focal SCNAs are associated with intrinsic resistance to AI treatment.

**CONTEXT**

**Key Objective**
Our study was focused on understanding the link between somatic copy number alterations (SCNAs) and intrinsic resistance to aromatase inhibitor (AI) therapy, and we observed that tumors with high levels SCNAs had intrinsic resistance to therapy.
**Knowledge Generated**
A well-established link exists between high genomic instability and *TP53* mutations and resistance to cancer treatment; however, we are the first to our knowledge to show that primary estrogen receptor (ER)–positive tumors with high genomic instability have an intrinsic resistance to treatment that can be measured after a short, 2-week AI treatment. High genomic instability tumors do not require time to evolve resistance to estrogen deprivation therapy because they already have de novo resistance to treatment.
**Relevance**
Estrogen deprivation therapy with AI treatment is highly effective in ER-positive breast cancer, but more than 20% of postmenopausal women with early-stage breast cancer suffer a relapse. The Perioperative Endocrine Therapy—Individualizing Care (POETIC) phase III trial with 2 weeks of perioperative AI therapy offers the opportunity to identify mechanisms and biomarkers of intrinsic AI resistance, and in the POETIC trial, up to 20% of tumors showed resistance to AI treatment after just 2 weeks of treatment. The results show that high genomic instability is associated with AI resistance, and detection of copy number alterations and mutations in *TP53* are predictive of high genomic instability. Validation of these results in a larger study would provide a framework for better stratifying patients into high risk of AI resistance who are likely to benefit from added or alternative treatment.

Response to AI treatment can be measured by change in the proliferation marker protein encoded by the *MKI67* gene (Ki-67) after 2 to 4 weeks of presurgical therapy, and AI resistance in primary tumors can be characterized and defined by limited or no Ki-67 response to AI treatment.^8-10^ This change in Ki-67 has been found to predict benefit from endocrine therapy better than clinical response.^10^ We therefore extended our earlier study on the relationship between mutations and resistance to AIs in the presurgical Perioperative Endocrine Therapy—Individualizing Care (POETIC) trial. We used single nucleotide polymorphism (SNP) array technology to identify SCNAs and included paired baseline and surgery samples to assess the degree of intratumoral heterogeneity and selection during AI treatment.

## PATIENTS AND METHODS

### Patients and Tissues

The POETIC trial (CRUK/07/015) is a presurgical, randomized study with 4,486 postmenopausal patients who received nonsteroidal AI (anastrozole 1 mg/d or letrozole 2.5 mg/d) or no treatment (2:1) 2 weeks before surgery.^11^ The list of primary investigators is in the Appendix. Core-cut biopsy specimens (14-G needle) were collected from 15% of patients into RNAlater (QIAGEN, Sussex, United Kingdom). Whole blood was collected for germline DNA and used as normal diploid control for SCNA analysis. The trial was approved by the London–South East Research Ethics Committee. Patients gave informed consent for DNA analysis.

### Biomarker Analyses

Ki-67 percent staining was centrally analyzed in formalin-fixed samples as previously described.^8^ Human epidermal growth factor receptor 2 (HER2) status was measured locally.

### Sample Selection

DNA was extracted from 192 baseline/surgery samples from the subset of POETIC ER-positive tumors stored in RNAlater and matching blood control samples from 73 patients with baseline Ki-67 scores greater than 5%. Poor responders (PrRs; n = 28) were defined as having a Ki-67 decrease of less than 60% between baseline and surgery and good responders (GdRs; n = 28) as having a greater than 75% Ki-67 decrease. Patients with intermediate Ki-67 decrease between 60% and 75% were not considered. Exome sequencing was available for 72 tumors from a previous study.^4^ Samples from 17 patients who received no AI also were analyzed to ensure that changes in SCNAs ascribed to AI treatment were not artifactual. Aliquots were taken from 10 tumor DNA samples and assessed as technical replicates (Data Supplement).

### DNA Extractions

Eight-micrometer sections were taken from RNAlater-stored core-cuts embedded in optimal cutting temperature compound (Cryo-M-Bed, Bright Instruments, Luton, United Kingdom) and stained with nuclear fast red (0.1% weight-to-volume ratio). Needle microdissection was used to achieve more than 60% pure tumor cells when necessary. DNA was extracted from the sections using a DNeasy Blood & Tissue Kit (QIAGEN) and from peripheral blood using the EZ1 system (Life Technologies, Carlsbad, CA).

### SNP Array Analysis

Human OmniExpressExome v.3 BeadChip (Illumina, San Diego, CA) was used to generate genotype and intensity data for blood and tumor samples, and allele-specific copy number analysis of tumors (ASCAT)^13^ was used for the estimate of ploidy, fraction of tumor cells, and CNAs in the tumor samples. Two samples did not pass OncoSNP quality control^14^ and visual inspection of the SNP array data. Ploidy and purity using default parameters and a range of higher segmentation penalties were estimated with ASCAT and OncoSNP. Either the segmentation penalty in ASCAT was increased (22 samples) or the estimate of ploidy and purity from OncoSNP was used in ASCAT (four samples) to generate SCNA calls that best described the data. For five samples, germline genotype predictions generated by ASCAT were the result of contamination or quality control failure of blood controls. BEDTools multi-intersect^15^ was used to identify 47,807 nonoverlapping segments from all samples. Data have been deposited in the European Genome-phenome Archive (EGAS00001001940).

### Measures of Genomic Instability

Chromosomal gains and losses were determined relative to estimates of tumor ploidy by ASCAT (sum of major and minor allele calls minus tumor ploidy rounded to the nearest integer). Loss of heterozygosity (LOH) was assigned when the estimated copy number was 0 for the minor allele. Genomic instability was defined as the percentage of the genome with SCNAs calculated by summing the total base pairs of segments with gain, loss, or LOH relative to paired normal blood control samples for each tumor sample and dividing by the size of the genome (3 × 10^9^ base pairs).

### Intrinsic Subtypes

Prediction analysis microarray 50 intrinsic subtypes were determined for 36 tumors.^12^ Details are listed in the Data Supplement.

### Statistical Methods

Mann-Whitney *U* test, *F* test, χ^2^ test, Pearson’s correlation, Fisher’s exact tests, and multiple correction by Benjamini-Hochberg method^16^ (false discovery rate [FDR]) were carried out using the wilcox.test, var.test, chisq.test, cor.test, fisher.test, p.adjust functions in R, respectively. Fisher’s exact tests were one-sided, and the remaining reported *P* values were from two-sided tests unless otherwise specified. Box plots were generated with the boxplot function in R to show median, interquartile range, and range of values, excluding outliers.

## RESULTS

### SCNA Characteristics in the Overall Population

**FIG 1.**
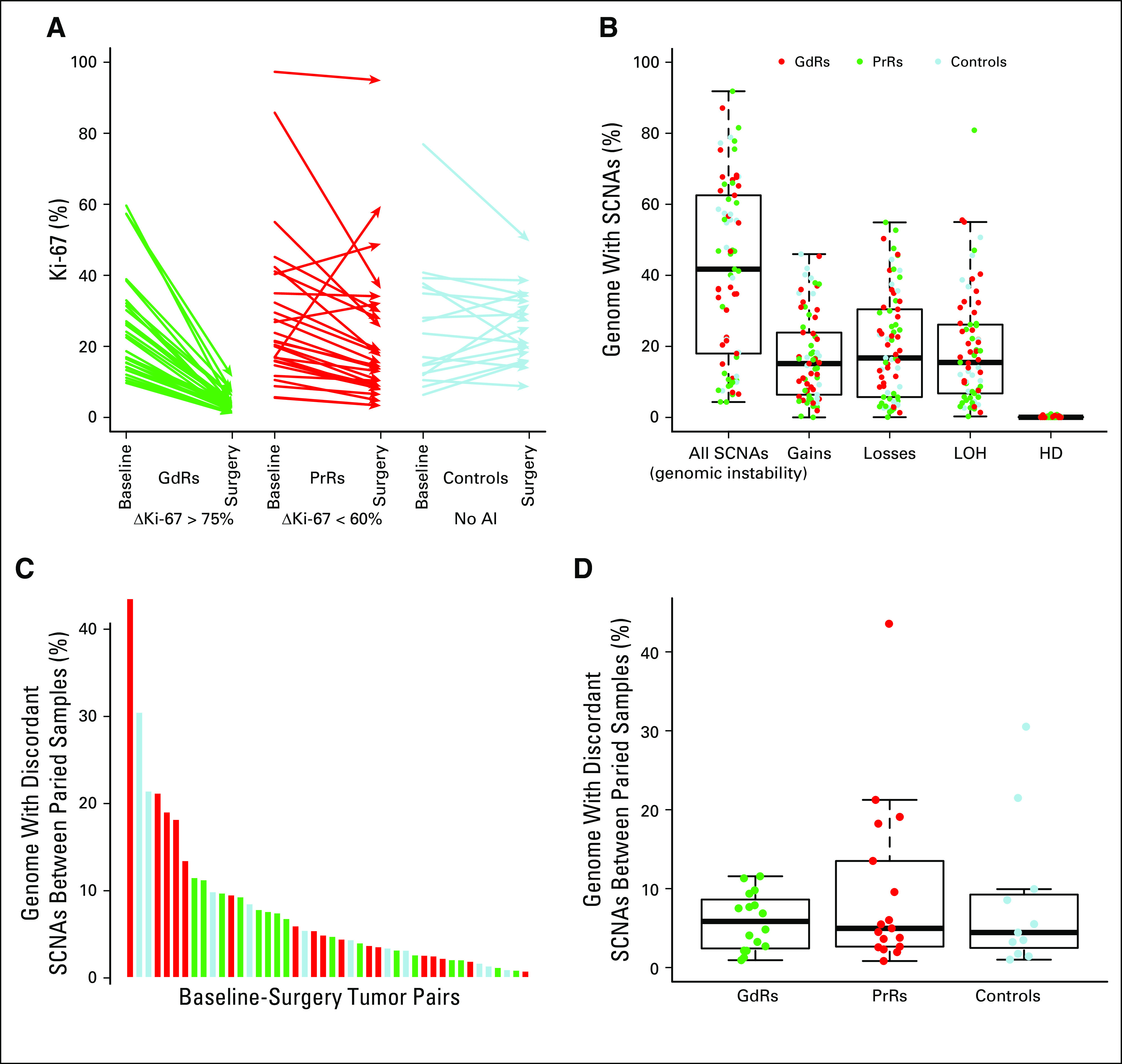
(A) Arrow plot showing the change in the protein encoded by the *MKI67* gene (Ki-67) between baseline and surgery for good responder (GdR), poor responder (PrR), and untreated control samples (Controls as determined by immunohistochemistry scores). (B) Box plot showing percentage of the genome with somatic copy number alterations (SCNAs), gains relative tumor ploidy, losses relative to tumor ploidy, loss of heterozygosity (LOH), and homozygous deletion (HD) for 127 tumor samples. (C) Bar plot and (D) box plot showing the average percentage of genome discordance between pairs of core-cuts (baseline and surgery) for all SCNAs. AI, aromatase inhibitor; IHC, immunohistochemistry.

SCNAs were identified in 28 patients with tumors classified as PrRs, 28 with tumors classified as GdRs, and 17 from the no-treatment control group with tumors (Fig 1A). The median percentage of the genome with SCNAs was 46% for all tumors, with a single representative tumor sample chosen from matched baseline, surgery, or technical replicate samples to calculate the median percentage of SCNAs. The median percentage of the genome with gains relative to tumor ploidy, losses relative to tumor ploidy, and LOH was 15%, 16%, and 15%, respectively (Fig 1B). Highly recurrent SCNAs (gains at 1q, 16p, 20q, and 8q and losses/LOH at 11q 16q, 17p, and 8p) occurred in more than 50% of all representative samples. The majority of sites with losses overlapped with LOH (Data Supplement), as expected.^17,18^

### Intratumoral Heterogeneity of SCNAs

#### Overlap of SCNAs between paired core-cuts.

Discordance between baseline and surgery time points was significantly greater than differences between technical replicate samples taken from the same DNA extraction (Data Supplement). Discordance in SCNAs was observed in more than 10% of the genome in only one pair of technical replicate samples. Of note, these samples had the highest genomic instability, with more than 90% of the genome with SCNAs (P088 samples).

Overall SCNA calls in baseline and surgery AI pairs were similar (Data Supplement), with the median overlap for SCNAs at 87% and 88% for 33 baseline/surgery AI pairs and 11 no-AI pairs. There was no significant difference between the frequency of discordant SNCA calls between baseline and surgery AI pairs after correction for multiple testing, and only 4% of 47,807 nonoverlapping regions had greater than 10% more events in baseline or surgery samples (more than four additional SCNA events in the baseline or surgery samples in the 33 pairs). Much larger sample sizes are required to determine whether these regions are significantly different between baseline and surgery.

#### Concordance of SCNAs between paired core-cuts.

For pairs of baseline and surgery samples, the median percentage of the genome with discordant SCNA calls was 5% (Fig 1C), and discordance between samples was associated with the percentage of the genome with SCNAs. There was only one paired set of core-cuts in which discordant SCNAs were greater than the SCNAs shared between the pair of samples, which suggests two independently evolved tumors.

#### Discordance in PrR and GdR paired samples.

There was a trend for PrRs to have more discordant SCNAs between paired samples than GdRs (PrR average, 10%; GdR average, 6%), but this difference was not significant. However, the variance in the percentage of the genome with discordant SCNAs was significantly greater in PrRs than in GdRs (*P* < .001, *F* test; Fig 1D). These data indicate that the tumors with the highest topographic heterogeneity in SCNAs were more frequent among the PrRs.

### Intrinsic Subtypes

Prediction analysis microarray 50 intrinsic subtype calls^12^ were performed on 36 baseline tumors. There was an enrichment of poor prognosis intrinsic subtypes (PrR nonluminal/luminal B) in PrR samples (64%) compared with GdR samples (20%); however, more than 30% of measured PrR samples were luminal A subtypes, which suggests that intrinsic subtyping did not fully capture the higher risk of recurrence in these samples (Data Supplement).

### Intertumoral Heterogeneity in SCNAs

#### Comparison between PrRs and GdRs in percentage of genome altered.

**FIG 2.**
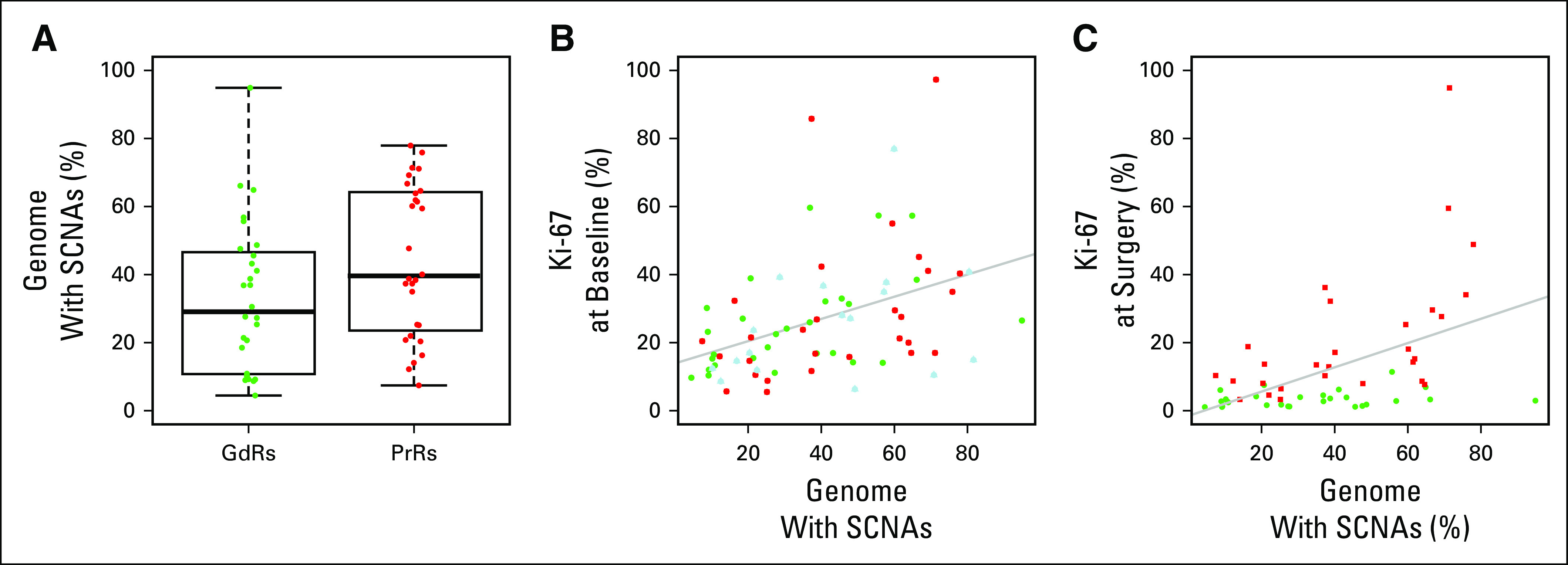
(A) Box plot showing the difference in genomic instability (the percentage of genome with somatic copy number alterations [SCNAs]) between good responder (GdR) and poor responder (PrR) tumors. (B) Comparisons of protein encoded by the *MKI67* gene (Ki-67) baseline immunohistochemistry scores with genomic instability (the percentage of the genome with SCNAs) for GdR, PrR, and untreated control samples (Controls, blue). (C) Comparisons of Ki-67 surgery immunohistochemistry scores after aromatase inhibitor treatment with genomic instability (the percentage of the genome with SCNAs) for PrRs and GdRs. Gray lines represent regression lines.

Given the overall concordance between baseline and surgery core-cuts in SCNAs and the results of previous observations of minimal impact of AI treatment on mutation counts,^4^ we merged all the SCNA events from multiple samples from the same tumor to represent the SCNA events in that tumor (baseline and surgery, 35 events; baseline, surgery, and technical replicates, nine events; baseline technical replicates, one event). The genomic instability was higher in the 28 PrR combined samples than the 28 GdR combined samples (*P* = .048, Mann-Whitney *U* test), and genomic instability was significantly correlated with baseline (*r* = 0.41; *P* < .001, Pearson’s correlation) and surgery (*r* = 0.48; *P* < .001; Pearson’s correlation) Ki-67 (Fig 2).

#### Comparison of SCNAs between PrRs or GdRs.

**FIG 3.**
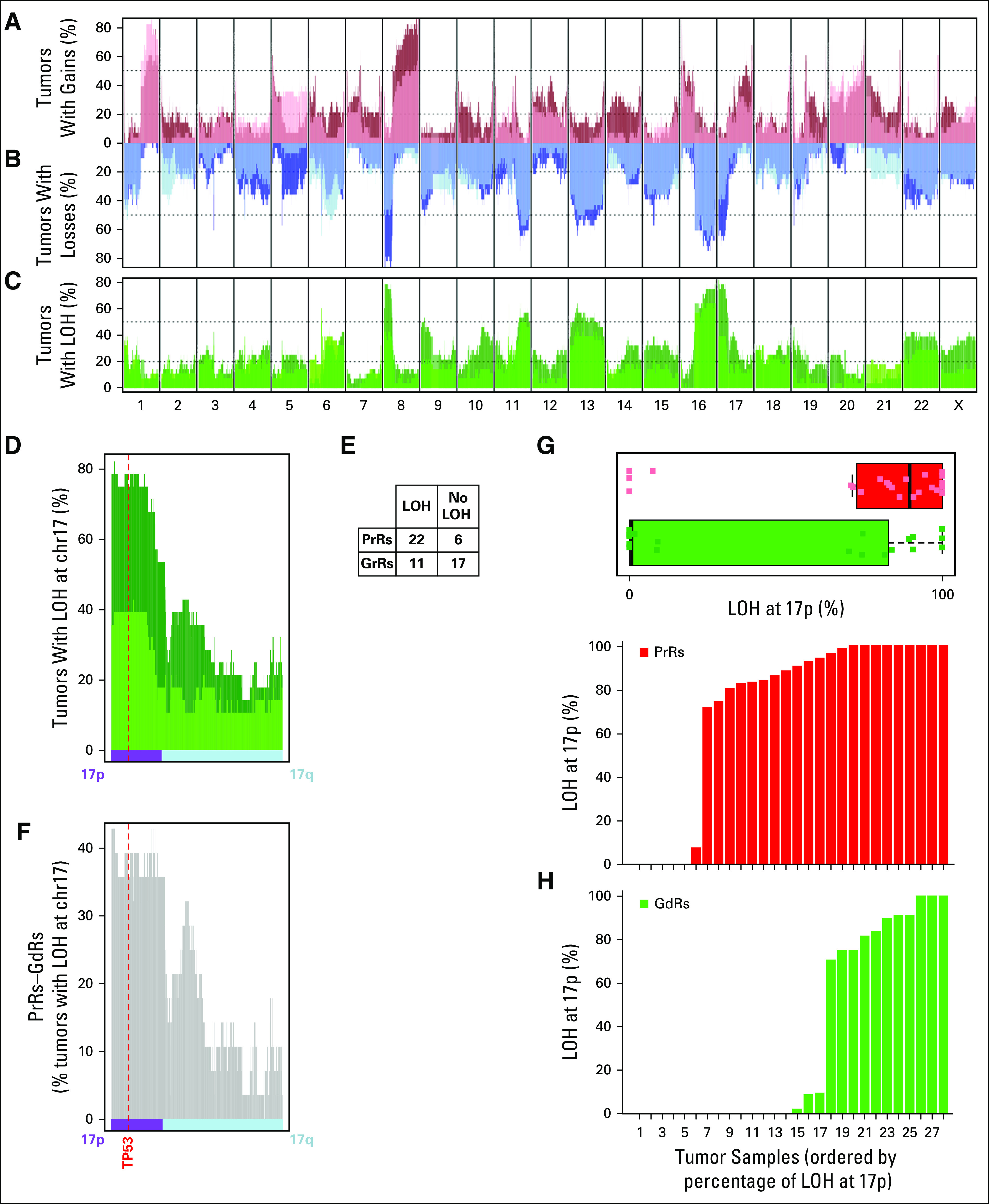
(A) Percentage of samples with gains relative to tumor ploidy for poor responders (PrRs; dark red) and good responders (GdRs; light red), (B) with losses for GdRs (light blue) and PrRs (dark blue), and (C) with loss of heterozygosity (LOH) for GdRs (light green) and PrRs (dark green) at 47,807 segments generated from the somatic copy number alteration (SCNA) analysis of Perioperative Endocrine Therapy—Individualizing Care (POETIC) tumor samples. (D) Percentage of samples with LOH (GdRs, light green; PrRs, dark green) for chromosome 17 (chr17), including (E) a table for LOH events at *TP53* and (F) the difference in the percentage of samples with LOH between PrRs and GdRs. (G) Box plots that show the percentage of 17p with LOH for GdRs (green) and PrRs (red) and (H) bar plots showing the percentage of LOH at 17p for each tumor.

The percentage of a chromosomal arm with gains, losses, and LOH was calculated, and PrRs showed a significantly higher percentage of gains in 6p; losses in 5q; and LOH in 10q, 17p, and 19p (FDR < 0.1, one-sided Mann-Whitney *U* test; Figs 3A to 3C). The largest difference in percent values (mean and median) for arms between GdRs and PrRs was observed in LOH at 17p (Figs 3D to 3G) followed by LOH in 8p and gains in 8q. There were no chromosomal arms with significantly greater gains, losses, or LOH in GdRs.

Analysis of smaller regions on the basis of the 47,807 nonoverlapping segments revealed that the most significant differences in gains were observed at 10p12.31 and 10p13 (*P* < .001, Fisher’s exact test), losses at 5q11.2 (*P* < .001), and LOH at 17p13.3 (*P* < .001). These regions had approximately 40% more events in PrRs (10 to 13 more SCNA events in the 28 PrR samples *v* GdR samples, respectively) but were not significant after multiple correction.

### *TP53* Alterations

#### Occurrence of *TP53*^MUT^ and LOH in cohort.

**FIG 4.**
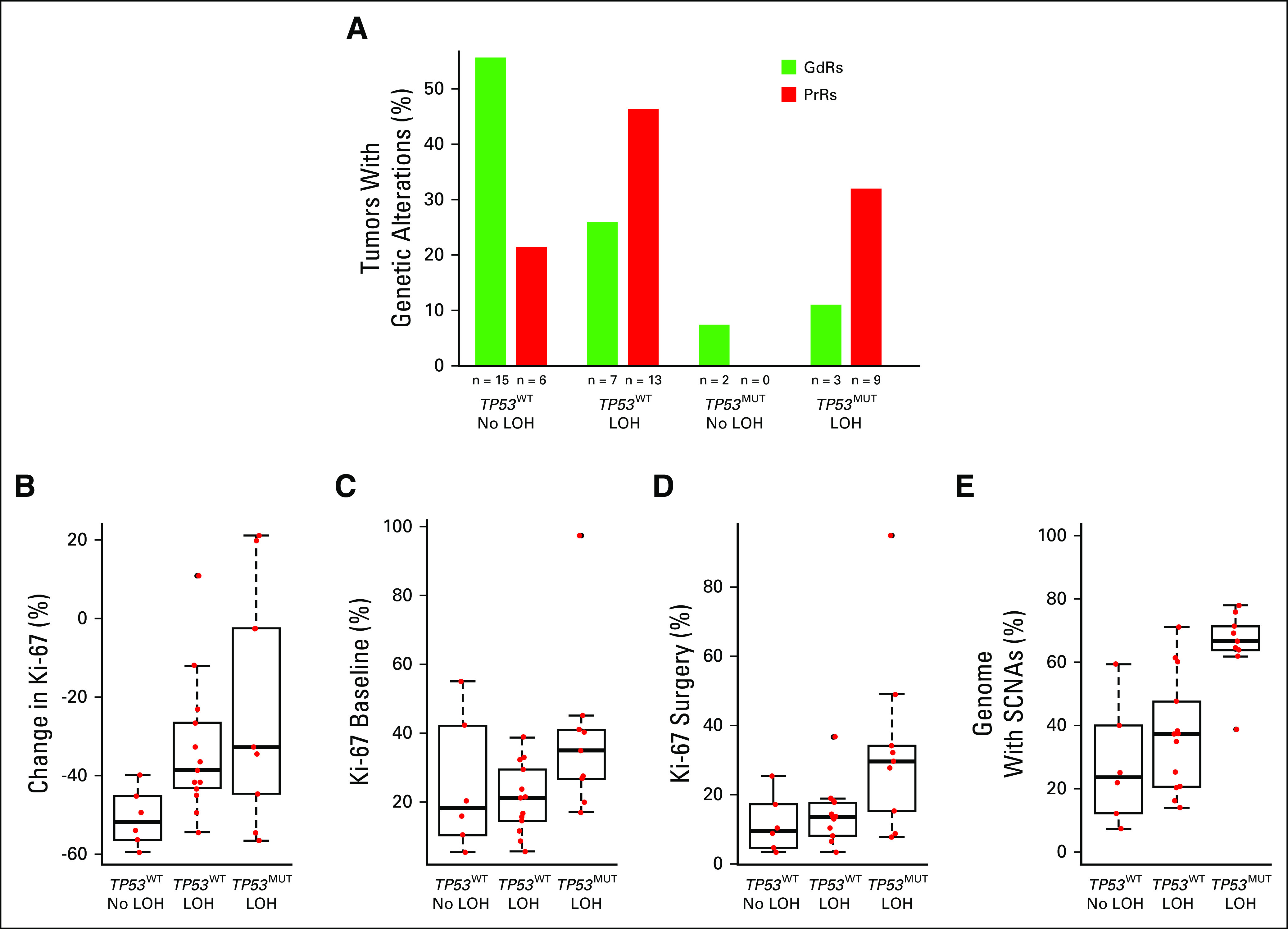
Bar plot showing the percentage of good responder (GdR) and poor responder (PrR) samples with *TP53* wild type (*TP53*^WT^) and no loss of heterozygosity (LOH) at the *TP53* locus, *TP53*^WT^ and LOH at the *TP53* locus, *TP53* mutation (*TP53*^MUT^) and no LOH at the *TP53* locus, and *TP53*^MUT^ and LOH at the *TP53* locus. Note that one GdR did not have exome sequencing data. Box plots showing (B) the percent change in the protein encoded by the *MKI67* gene (Ki-67), (C) the Ki-67 baseline immunohistochemistry scores, (D) the Ki-67 surgery immunohistochemistry score, and (E) genomic instability (the percentage of the genome with somatic copy number alterations [SCNAs]) for PrRs with *TP53*^WT^ and no LOH at the *TP53* locus, *TP53^WT^* and LOH at the *TP53* locus, and *TP53*^MUT^ and LOH at the *TP53* locus. There were no PrR samples with *TP53*^MUT^ and no LOH at the *TP53* locus.

Our previous work from exome sequencing showed PrRs and *TP53*^MUT^ associated with a higher mutational load and that the mutational load was correlated with Ki-67 levels at surgery after 2 weeks of AI treatment.^4^ We did not observe a significant correlation between the percentage of the genome with SCNAs and mutational load, but we did observe greater genomic instability in tumors with *TP53*^MUT^ (Fig 4E).

As expected for a tumor suppressor, LOH at the TP53 locus in 17p was associated with TP53^MUT^ across all tumors (which drove loss of the functioning copy of the tumor suppressor gene; *P* = .004, Fisher’s exact test). Of the 17 patients with *TP53* mutations in baseline or surgery samples, 15 had LOH at the *TP53* locus (nine PrR, five GdR, and three control samples). All nine PrR samples and three of five GdR samples with *TP53*^MUT^ also had LOH at the *TP53* locus. There was a significant enrichment of TP53 genomic alterations in PrRs (*P =* .03, Fisher’s exact test) and a significant difference in the distribution of *TP53* genetic alterations between PrRs and GdRs (*P =* .02, χ^2^ test; Fig 4A).

#### AI resistance and TP53 status.

Within the PrR group, samples with no LOH and *TP53* wild type (*TP53*^WT^) had the best antiproliferative response to AI compared with samples with *TP53*^WT^ + LOH and *TP53*^MUT^ + LOH as measured by the change in Ki-67 (*P* = .01 and .05, respectively, Mann-Whitney *U* test; Fig 4B). The difference in the change in Ki-67 between *TP53*^WT^ + LOH and *TP53*^MUT^ + LOH was not significant, but there were significant differences between *TP53*^WT^ + LOH and *TP53*^MUT^ + LOH for baseline Ki-67 scores (*P* = .02) for surgery Ki-67 scores (*P* = .04) and for the percentage of the genome with SCNAs (*P* < .001; Figs 4B to 4E).

#### Impact of HER2 status.

There were seven HER2-positive samples in the PrR group and none in the GdR group. HER2-positive samples had a significantly higher percentage of the genome with gains in copy number compared with HER2-negative PrR samples (*P* = .03, Mann-Whitney *U* test) but did not have a significantly higher percentage of SCNAs in general, losses, or LOH. The results with HER2-negative samples were similar to those with all samples, with the most significant differences between PrRs and GdRs being loss at 5q and LOH at 17p for HER2-negative samples. There was also a significant enrichment of TP53 genomic alterations in PrRs (*P =* .02, Fisher’s exact test) and a significant difference in the distribution of *TP53* genetic alterations between PrRs and GdRs in HER2-negative samples (*P =* .03, χ^2^ test).

## DISCUSSION

Our primary goal was to identify global and focal SCNAs that were associated with the antiproliferative response of ER-positive BC to short-term estrogen deprivation using AIs. Our selection of samples from more than 3,000 patients in the AI group from the POETIC trial aimed to exploit this large study to understand good/poor response to AI treatment in a general ER-positive BC population but not to represent the trial population per se. The sampling of tumors before and after 2 weeks of AI treatment allowed the impact of tissue heterogeneity to be assessed, and prior exome sequencing gave the opportunity to integrate the SCNA and mutation data to better understand intrinsic resistance. Although the number studied seems modest, the ability to assess response in individual tumors allows much greater confidence with molecular associations than larger studies with time to recurrence. HER2 positivity was enriched in the PrRs, as previously noted,^4^ but the genomic changes were similar in HER2-negative patients and the overall population.

The lack of recurrent alterations specific to only baseline or surgery in AI-treated samples indicates a limited impact and selection for SCNAs after 2 weeks of AI treatment, in line with other studies.^4,19^ Of note, mean tumor volume did not change significantly in the nearly 3,000 POETIC AI-treated patients within the 2-week treatment window (data not shown), which indicates little opportunity for selection of resistant cells in that time. Reduced heterogeneity might be observed from longer treatment.^20^ These data, therefore, indicate that a small biopsy sample before or after short-term AI treatment is likely to be representative of the whole tumor for most BCs; however, for tumors with high genomic instability and greater heterogeneity, multiple biopsy samples may be necessary to capture all genomic alterations.

There is a large body of evidence to associate genomic instability with poor outcomes in solid tumors,^6^ and incorporation of genomic instability scores can greatly improve molecular prognostic models for BC.^21,22^ It is not known whether high genomic instability and greater tumor heterogeneity allow the few surviving tumors to evolve resistance to AI treatment or whether there is intrinsic resistance to AI in these tumors. Our data support the latter, with tumors with high genomic instability showing de novo resistance to AI therapy as measured by a poor Ki-67 response after 2 weeks of treatment, a validated intermediate marker of benefit from endocrine therapy.^10^ This also suggests that genomic instability not only has prognostic value but also predicts which postmenopausal ER-positive primary tumors are likely to be resistant to AI therapy.

The SCNA LOH in 17p was significantly associated with poor Ki-67 change, and LOH was significantly greater in PrRs than in GdRs in HER2-negative tumors and the overall population. This region encodes for several cancer driver genes, including *TP53*, a key regulator of cellular processes that control proliferation and genomic stability. LOH and mutations in *TP53* have been shown to result in worse outcomes,^23^ and we have now shown that it is also associated with poor antiproliferation response to AI and intrinsic resistance to treatment. Other factors besides *TP53* can modulate genomic instability and AI resistance, and genomic instability is significantly inversely correlated with the average expression of the ER-regulated genes *TFF1*, *GREB1*, *PGR*, and *PDZK1* in ER-positive tumors from the Molecular Taxonomy of Breast Cancer International Consortium^24^ (*r* = −0.24; *P* < .001, Pearson’s correlation), which suggests that other factors besides ER are driving proliferation and resistance to AI in tumors with high genomic instability. Even in tumors with high ER expression and good prognosis, *TP53* genomic alterations can result in worse outcomes.

Work by other groups has associated mutations in DNA repair pathways^25^ or mismatch repair pathways^19^ and co-amplification of *FGFR1* and *CCND1*^5^ with resistance to AI treatment, but we have not observed enrichment of these genomic alterations in our PrRs. This may be the result of small samples sizes in each study and additional differences in how AI resistance is classified: We classified response/resistance on the basis of changes of Ki-67 between baseline and AI-treated tumors because this dynamic assessment relates to benefit from treatment. Others have used the level of residual Ki-67 in AI-treated tumors as the end point to define resistance, which reflects residual risk of recurrence while on AIs. Of note, a patient with a large reduction in proliferation after treatment has clearly benefited from and responded to AI treatment, regardless of her residual risk on the basis of Ki-67 measurements at surgery.^26^

We conclude that the poor prognosis of ER-positive postmenopausal tumors associated with high genomic instability, *TP53* LOH, and *TP53*^MUT^ is due at least in part to intrinsic resistance of these tumors to AI therapy. Short, 2-week AI treatment can reveal poor antiproliferative response in these primary tumors, which indicates that they continue to proliferate in an estrogen-deprived environment and do not require additional evolution to enable the tumor to resist treatment. It is not clear whether high genomic instability or *TP53* genomic alterations directly play a role in AI resistance or whether these are biomarkers for other drivers of resistance. Additional analysis of the more than 3,000 AI-treated patients from POETIC may reveal additional links among genomic instability, *TP53*, and AI resistance and lead to better treatment of those patients with high genomic instability and intrinsic resistance to AI treatment.

## Appendix

POETIC Trial Members
